# Nutritional Value of the Larvae of the Alien Invasive Wasp *Vespa velutina nigrithorax* and Amino Acid Composition of the Larval Saliva

**DOI:** 10.3390/foods9070885

**Published:** 2020-07-06

**Authors:** Hyeyoon Jeong, Ja Min Kim, Beomsu Kim, Ju-Ock Nam, Dongyup Hahn, Moon Bo Choi

**Affiliations:** 1 School of Food Science and Biotechnology, College of Agriculture and Life Sciences, Kyungpook National University, Daegu 41566, Korea; ddi02084@naver.com (H.J.); kimjamin1987@naver.com (J.M.K.); vincent1580@naver.com (B.K.); namjo@knu.ac.kr (J.-O.N.); 2 Institute of Agricultural Science and Technology, Kyungpook National University, Daegu 41566, Korea; 3 Department of Integrative Biology, Kyungpook National University, Daegu 41566, Korea; 4 School of Applied Biosciences, College of Agriculture and Life Sciences, Kyungpook National University, Daegu 41566, Korea

**Keywords:** edible insects, alternative food resource, wasp larva, *Vespa velutina nigrithorax*

## Abstract

The systematic investigations on the value of social wasps as a food resource are deficient, in spite of the long history of the utilization of social wasps as food and pharmaceutical bioresources. *Vespa velutina nigrithorax* is an invasive alien wasp species that is currently dominating in East Asia and Europe, bringing huge economic damages. As a control over alien species is made when the valuable utilization of the invasive species as a potential resource are discovered, investigations on the potential of *V. v. nigrithorax* as a useful bioresource are also in demand. Nutritional and heavy metal analyses of the larvae revealed their balanced and rich nutritional value and safety as a food resource. The larval saliva amino acid composition was investigated for further study on amino acid supplementation and exercise enhancement.

## 1. Introduction

Social wasps are recognized as harmful insects. They are a threat to humans due to their stinging behaviors that might lead to death [1,2] and their aggressive nature to hunt for honeybees brings huge economic losses in the beekeeping industry [1,3,4]. However, especially in East Asia, social wasps are bioresources that have been utilized for medicinal and food purpose for a long time. In Korea and China, nests of social wasps (*Nidus vespae*) have been used as an ingredient of crude drugs [5,6], or an ingredient of health-promoting traditional liquors [5]. The entomophagic records about wasps are mostly related to consumption of larvae, which are available in Papua New Guinea [7], China [8] and Laos [9]. In Japan, larvae of wasps, ‘*hachinoko*’ in Japanese, have long been consumed, and even canned products of the larvae are available at markets [10]. However, wasp larvae have been rather delicacies—not part of daily diets—due to their limited supply from nature. Particularly in China and Japan, where wasp materials provided by wasp hunters are traded at high prices and where wasps were traditionally used as food or pharmacological resources, there have been attempts to develop wasp breeding techniques to ensure sufficient supply of materials [8,11]. Fortunately, in some cases, embryo nest breeding was often successful, but none of the reliable systematic breeding technology has been established yet [8]. To establish wasp larvae as a regular food resource, techniques for mass rearing to ensure sufficient quantities of materials is required [12]. However, prior to the supply issue, it is necessary to evaluate if wasp larvae are worth development for a regular food resource in regards to their nutritional value and food safety. The investigation on *Vespa mandarinia* larvae by Kim and Jung [13] in 2013 is the only attempt to evaluate the nutritional value of wasp larvae to investigate potential as a food material including taxonomic identification.

Invasion of *V. v. nigrithorax* is a momentous ecological issue to Europe and East Asia now. Since the first invasion of *V. v. nigrithorax* into South Korea in 2003, it has spread throughout the country [1,5,14,15,16], and invaded Europe [17] and Japan [18]. *V. v. nigrithorax* have a serious economic impact as they hunt honeybees in apiaries [2,19] and a public health impact as they sting people in urban areas [1]. This wasp is also causing ecosystem disturbances through competition and interference among *Vespa* species within the ecosystem [19,20,21]. The southern region of Korea is densely populated by *V. v. nigrithorax*, and this wasp is the most prevalent in urban and forest areas [22]. In this context of momentous significance, *V. v. nigrithorax* was selected for investigation.

In this study, we collected larvae from *V. v. nigrithorax* nests and examined the amino acid composition of their saliva, and nutritional analyses of larvae were performed. The nutritional value of *V. v. nigrithorax* larvae have not been revealed, though *V. v. nigrithorax* is now one of the frequently encountered species in East Asia.

## 2. Materials and Methods

### 2.1. Wasp Larvae Collection and Preparation

**Figure 1:**
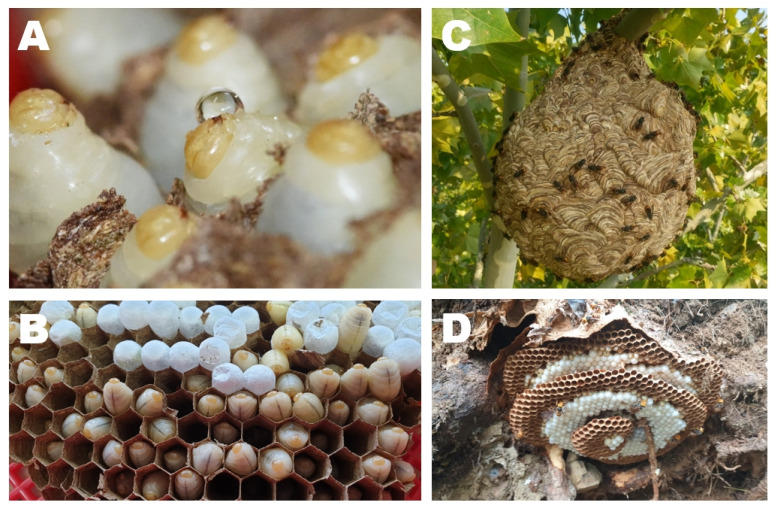
The nest of *V. v. nigrithorax* that hovers larvae. (**A**) Larvae of *V. v. nigrithorax* secreting saliva; (**B**) larvae of *V. v. nigrithorax* in cells; (**C**) the nest of *V. v. nigrithorax* hung on a tree; (**D**) the nest of *V. mandarinia.*

Two *V. v. nigrithorax* nests were collected from tree branches at heights of approximately 10–15 m in October 2018 in Daegu, South Korea (Figure 1). One of the authors (M.B.C.) identified the builders of these nests as *V. v. nigrithorax*. The nests contained mature colonies with approximately 1200–1400 adults. The adults were removed from the nests and the combs were separated. Healthy larvae of the fourth or fifth stage were selected for saliva collection. The rest of the larvae were preserved at −80 °C for nutritional analysis.

### 2.2. Larval Saliva Preparation

To induce salivation, capillary pipette tips were used to gently tap the mouth parts of the larvae and collect saliva. A 50–200 μL volume of clear saliva was collected from every larva. The tubes containing saliva were immediately frozen and kept at −80 °C until analysis.

### 2.3. Amino Acid Analysis of the Larvae and Larval Saliva

Qualitative and quantitative analysis for the composition of amino acids in larval saliva was performed through the amino acid automatic analyzer (L-8900, Hitachi, Tokyo, Japan) equipped with an ion exchange column (No. 2622 SCPF, 4.6 mm × 60 mm). Buffer solutions PF-1, 2, 3, 4, 6, PF-RG, R-3, and C-1 (Wako, Osaka, Japan) were used as the mobile phase with a flow rate of 0.35 mL/min, and the column temperature was maintained at 50 °C. The reaction liquid flow rate was 0.3 mL/min, monitoring the wavelengths of 440 nm from 570 nm, and the injection volume was 20 μL. The concentration of amino acids in larval saliva samples was determined by the peak area of standard samples. For hydrolyzed amino acid analysis, 1 g of freeze-dried larvae and 40 mL of 6 N HCl were placed in a round flask and mixed, followed by hydrolysis by injecting nitrogen gas at 110 °C for 24 h. The hydrochloric acid was concentrated under reduced pressure at 50 °C, and the concentrated sample was diluted with 50 mL of 0.2 N sodium citrate buffer (pH 2.2) and filtered with filter paper (0.45 μm). The filtered sample (30 μL) was analyzed using an amino acid analyzer (L-8900, Hitachi, Tokyo, Japan).

### 2.4. Nutirtional Analysis of the Larvae

The nutritional composition (ash, moisture, lipid, protein and carbohydrate) of lyophilized larvae was determined by following the standard method of the Korean Food Standard Codex [23]. Briefly, the crude protein content was determined by the Kjeldahl method using a crude protein analyzer (Kjeltec™ 8400, Foss, Hilleroed, Denmark), and was calculated by multiplying the nitrogen content using a coefficient of 6.25. Crude fat was extracted from larvae in a Soxhlet apparatus with ethyl ether as the solvent. Moisture content was determined by drying the sample in an oven at 105 °C until a constant weight was obtained. Ash content was determined by dry-ashing in a furnace (J-FM3, Jisico, Seoul, Korea) at 550 °C for 12 h. Carbohydrate content had been determined by subtracting the sum of the weights including ash, moisture, protein and lipid.

### 2.5. Hazardous Heavy Metals Ananlyses of the Larvae

The contents of hazardous metals, arsenic (As), cadmium (Cd), mercury (Hg) and lead (Pb) were determined using an inductively coupled plasma atomic emission spectroscopy instrument (ICP/OES, Avio 500, Perkin-Elmer, Waltham, MA, USA). For analysis, 140 mg of the lyophilized larvae underwent a nitric acid-pretreatment and were digested with a microwave digestion system (UltraWAVE, Milestone, Sorisole, Italy). The digested samples were diluted with 2% nitric acid (w/w) and injected for instrumental analysis. The analytical condition used for ICP/OES is described in Table 1.

## 3. Results and Discussion

### 3.1. Amino Acid Composition of Larval Saliva

One of a few examples for the application of wasp materials to food is the development of a supplementary beverage for physical exercise inspired from the amino acid composition of *V. mandarinia* larval saliva [24]. The beverage products do not contain the actual larval saliva of *V. mandarinia,* but an amino acid mixture composed of the same amino acid composition of *V. mandarinia* larval saliva. The products were developed on the basis of a series of investigations on exercise endurance enhancement effects by supplementation of the amino acid mixture of which the composition is the same as the larval saliva of *V. mandarinia* [25,26,27]. This idea has come from trophallaxis, a unique way of survival observed in social wasps. Trophallaxis is common among wasps; carnivorous larvae which feed on meat pellets prepared by adult workers, while larvae produce and pass an oral exudate to adult workers in response [27,28]. For social wasps, larval saliva is considered a source of energy and motivation for adult workers that fly a distance of about 100 km daily only to prepare meat pellets for larvae [29]. Takashi and colleagues reported the composition of amino acids in the larval saliva from five species of hornets including *Vespa mandarinia, Vespa crabo, Vespa tropica, Vespa analis* and *Vespa xanthoptera,* collected in central Japan, which has a temperate climate [27]. Based on this research, other related researches were performed subsequently, which were focused on observations of the metabolism of animals administered an amino acid mixture of an identical composition to the larval saliva of *V. mandarinia* [25,26,29,30,31,32]. The vespa amino acid mixture (VAAM) was coined, based on the free amino acid composition of the larval saliva of *V. mandarina*, which may be related to aggressiveness and the longest distance flown for hunting [25,27,29]. Studies on the metabolism using of exercising animals revealed that amino acids in larval saliva may act as energy sources and enhance exercise performance through controlling the metabolism [25,26,29,30,31]. *Vespa mandarinia*, the most dominant hornet species in central Japan, has been used as a reference, as it boasts the longest flight distance and the most extensive hunting domains [25,27,29].

Reports on *V. v. nigrithorax* invasions to temperate regions sparked an increasing interest in them as their distribution is expanding [1,16,33,34]. However, studies on trophallaxis and the larval saliva of *V. v. nigrithorax* has not been performed despite the significance of these topics. According to previous research by Takashi et al. [27], each wasp species has a peculiar larval saliva amino acid composition and larval saliva is the principal component of trophallaxis in hornets. There may be a number of factors that influenced the expansion of the *V. v. nigrithorax* territory beyond the tropical region. In addition, *V. v. nigrithorax* are found in numbers of 1500–2500 individuals per nest, and their nests are 2–3 times larger than those of other *Vespa* spp [2,35,36]. The amino acid contents in the larval saliva of *V. v. nigrithorax* might be correlated with the energy required for exercise and metabolism and could have influenced the growth of their population, which might inspire us to coin a supplementary diet formula. Thus, the amino acid composition of the larval saliva of *V. v. nigrithorax* was investigated.

As *V. mandarinia* has been the standard for the physiological studies involved in vespa amino acid mixture (VAAM) supplementation, our results on the amino acid composition of the larval saliva of *V. v. nigrithorax* were also presented alongside the amino acid composition of the larval saliva of *V. mandarinia* reported by Takashi et al. [27] (Table 2). A notable feature of the amino acid composition of the larval saliva of *V. v. nigrithorax* was the high proportion of the physiological amino acids compared to the total amount of amino acids measured from the hydrolyzed product of larval saliva. The total amount of physiological amino acids in the larval saliva of *V. v. nigrithorax* was 38.29 μmol/mL and the total amount of amino acids deduced from the hydrolyzed product was 67.24 μmol/mL; these results suggest that the larval saliva of *V. v. nigrithorax* has a higher ratio of free amino acids than that of *V. mandarinia*. Physiological amino acids are valuable nutrients for adult wasp workers; adult wasps are able to consume only liquid foods and hardly digest polymer-type nutrients, owing to their short and simplified gut structure and narrow waists [37]. Worker wasps in particular, spend most of their time flying for hunting purposes; therefore, free amino acids in larval saliva could help them prolong their flight time, as has been reported in previous studies on animal exercise physiology and VAAM supplementation [25,29]. The amino acids that composed the physiological amino acid mixture of the larval saliva of *V. v. nigrithorax* were similar to those of saliva from other wasps, as revealed by Takashi et al. [27]. A notable feature of the physiological amino acid composition of the larval saliva of *V. v. nigrithorax* was the high proportion of proline. The content of proline is believed to be correlated with the daily flight distance and hunting domain size of *Vespa* spp. [27], as proline is metabolized by insects to produce energy for hunting [38]. Branched chain amino acids (BCAAs) such as Ile, Val, and Leu were detected in substantial amounts (3.28, 5.45, and 3.75 mol%, respectively). In animals, BCAAs are directly metabolized in muscles as energy sources and the intake of BCAAs helps prevent muscular loss caused by endurance exercise [39]. The other essential amino acids (threonine, phenylalanine, lysine, and histidine) were detected in fair amounts (5.48, 5.34, 9.88, and 4.28 mol%, respectively), except for tryptophan and methionine.

The amino acid content of the hydrolyzed product of the larval saliva of *V. v. nigrithorax* revealed that a few amino acids were present as peptide constituents (Table 3). Acidic amino acids aspartate and glutamate were detected in significantly different amounts in the physiological and hydrolyzed amino acid mixtures. Aspartate and glutamate were detected in amounts of 0.06 and 1.71 μmol/mL, respectively, in the physiological amino acid mixture, while they were detected in amounts of 3.21 and 10.71 μmol/mL, respectively, in the hydrolyzed amino acid mixture. Other amino acids such as glycine, alanine, isoleucine, and lysine were detected in the hydrolyzed amino acid mixture in quantities that were about 2–3 times higher than those in the physiological amino acid mixture. The larval saliva of *V. v. nigrithorax* did not contain higher amino acid and protein amounts than that of *V. mandarinia*. Takashi et al. [27] suggested that high amino acid and protein contents in larval saliva may be correlated with the body weight and the ecological dominance of wasps in nature. However, the larval saliva of the invasive alien species *V. v. nigrithorax* has not been subjected to amino acid analysis up to this point.

### 3.2. Nutritional Composition of the Larvae

Table 4 represents the nutrient composition of the lyophilized *V. v. nigrithorax* larvae compared to other representative edible insect larvae, *Protaetia brevitarsis seulensis* and *Tenebrio molitor*. The nutrient compositions of *V. mandarinia, P. b. seulensis* and *T. molitor* larvae were quoted from previous reports [35,36,37]. Among carbohydrates in the larvae, the sugar content was 6.08 g/100 g. And the content of saturated fat was 4.31 g/100 g, while the content of trans fat was detected under the limit of quantification. Total cholesterol content was 31.97 mg/100 g. Sodium content was 60.91 mg/100 g.

The content of protein in *V. v. nigrithorax* larvae was similar to that of *T. molitor* larvae, and less than in *P. b. seulensis* larvae. The content of fat in *V. v. nigrithorax* larvae was slightly less than in *P. b. seulensis* larvae and far less than in *T. molitor* larvae. Interestingly, carbohydrate content in *V. v. nigrithorax* larvae was nearly three times of the content in other larvae. Overall nutrient contents in *V. v. nigrithorax* larvae imply the larvae may provide a nutritionally balanced diet, as they contain sufficient and balanced amounts of essential nutrients compared to other representative edible insect larvae, *P. b. seulensis* and *T. molitor*. Compared to dry whole milk, the protein content in *V. v. nigrithorax* larvae (48.64%) was an overwhelmingly great amount, as protein takes 26.3% of dry milk weight [42]. The protein content in *V. v. nigrithorax* larvae was even more than commercial dried whey protein concentrate products (protein contents 35–39%) [43].

In 2010, the South Korean government enacted the “Act of Fosterage and Support of the Insect Industry” to establish a legal basis for supporting the growth of the insect industry [44]. Along with the approved insect food ingredients (crickets, *T. molitor* larvae, *P. b. seulensis* larvae, and silkworm), wasps are often ingested in Korea even if they are not approved as food ingredients yet [13,45]. However, the nutritional analysis may provide firm grounds for approval of wasp larvae as a food ingredient along with the entomophagic records of wasp larvae in Japan [46], Papua New Guinea [7] and Laos [9].

### 3.3. Amino Acid Composition in Larvae

Composition of free amino acids in the larvae and amino acids in the hydrolyzed products of the larvae (Table 5.) demonstrated that the larvae of *V. v. nigrithorax* might be a balanced source for essential amino acids. Total content of free amino acids was nearly a quarter of constitutional amino acids of the larvae, which reflected that the larvae might be good resources for amino acid supplement. Twenty-seven amino acids were found in the free amino acid mixture, and sixteen amino acids were detected in hydrolyzed products of the larvae. In particular, essential amino acid contents, except Met and Trp (Arg, His, Ile, Leu, Lys, Phe, Thr and Val), were detected in substantial amounts in hydrolyzed products of the larvae (7.74–24.86 mg/g).

The amount of each hydrolyzed amino acid reflects that *V. v. nigrithorax* larvae could be a good supplementation for essential amino acids. Compared to the guideline of the World Health Organization (WHO) for daily adult essential amino acid requirement [47], the essential amino acids that might be provided by *V. v. nigrithorax* larvae are balanced and sufficient. Table 6 represents daily adult amino acid requirement suggested by the WHO, the amount of each essential amino acid required for an adult of 70 kg weight, and the amount of each essential amino acid equivalent to amino acid contained in 100 g of the larvae. On the basis of the guideline from the WHO, the essential amino acid requirements of a 70 kg adult was deduced, and could be mostly fulfilled by supplementation of amino acids provided from *V. v. nigrithorax* larvae. The amounts of Thr, Cys, Phe, Tyr, Lys, and His contained in 100 g of the larvae were more than the daily amino acid requirements for a 70 kg adult (104–143% of the requirement). Val and Ile provided by 100 g of the larvae were estimated to account for 99% and 97% of the requirements, respectively. The Leu content in 100 g of the larvae was 80% of the requirement. Except Trp and Met which were not detected in the larvae, the majority of the essential amino acids in the larvae are sufficient to fulfil the daily requirements of amino acids in adults.

### 3.4. Hazardous Heavy Metal Analysis

By the analysis using the ICP/OES instrument, contents of As, Pb and Cd were not detected, while 23.56 ppb of Hg was detected in the lyophilized larvae. Compared to other edible insects, wasp larvae have an extraordinary carnivorous nature; they are fed on smaller insects hunted by adult wasps and adults are fed on saliva of the larvae. In nature, it is common to contain more heavy metals in the body if an animal species is a higher predator. However, hazardous heavy metal contents in the larvae were not as considerable as a carnivorous animal which might accumulate heavy metals from prey. There is no regulation legislated by the Ministry of Food and Drug Safety (formerly Korea Food & Drug Administration, KFDA)for edible insect products concerning heavy metal contents yet. For fishery products, up to 500 ppb of Hg is allowed to be detected, therefore, the amount of Hg in the larvae would not harm consumers.

### 3.5. General Discussion

The habitat of *V. v. nigrithorax* is quickly expanding northward, particularly in Korea [48], since its first reported appearance in 2003 [16]. *V. v. nigrithorax* has serious negative public health, economic, and ecological impacts [1]. In recent years, invasion of alien species has gradually increased in Korea. However, in most of the cases, the controls over the invasive species were not successful, and various damages are rapidly increasing through the nationwide spread [49,50,51,52]. However, in some cases, the control of alien species is made when the valuable utilization of the invasive species as a potential resource are discovered [53]. *V. v. nigrithorax* was designated an ecological disturbance species in 2019, causing comprehensive impact in Korea, but the attempts to discover the utilization as a useful bioresource from this species are rare [54].

Owing to environmental pressures, food and feed insecurity, and a growing demand for protein, the consumption of insects to ease the problem of global food shortages has been advocated since 1975 [55]. Along with the approved insect food ingredients (crickets, mealworm, *Protaetia brevitarsis* larvae, and silkworm), wasps are also often ingested in Korea even if they are not yet approved as food ingredients by KFDA. The history of utilizing the social wasps as a food and pharmaceutical resources suggests that *V. v. nigrithorax* could be a valuable bioresource.

## 4. Conclusions

From this study, the potential of *V. v. nigrithorax* larvae as a food resource has been exhibited. They contain decent amount of nutrients, while hazardous metal contents are scarce. However, additional investigations in sanitary issues and sustainable production technology are necessary to accept the wasp larvae as an alternative food source. The larval saliva of *V. v. nigrithorax* will not provide original food sources, but the amino acid contents of it can be mimicked and might inspire the development of amino acid supplement formulas for exercise enhancement. This study will initiate future systematic investigations on the potential of *V. v. nigrithorax* as a valuable bioresource.

## Figures and Tables

**Table 1:** Analytical condition used for inductively coupled plasma atomic emission spectroscopy instrument (ICP/OES).

Condition	
Parameter	Specification
Power	1500 W
Plasma gas flow	10 L/min
Auxiliary gas flow	0.2 L/min
Nebulizer gas flow	0.6 L/min
Nebulizer	Concentric nebulizer, glass
Torch	Quartz
Spray chamber	Cylconic spary chamber
Injector	Alumina, inner diameter 2 mm
Pump tubing	Polyvinyl Chloride (PVC)
Sample pump flow	1.5 mL/min
Rinse/read delay	60 s
Integration time	3 (3 replicates)
Plasma view	Axial

**Table 2:** Physiological amino acid compositions of larval saliva of *V. v. nigrithorax* compared to *V. mandarinia.*

Amino Acids	*V. mandarinia*	*V. v. nigrithorax*	*V. mandarinia*	*V. velutina*
	(μmol/mL) ^a^	(μmol/mL)	(mol%) ^a^	(mol%)
phosphoserine	-	0.08	-	0.22
Tau	0.39	0.82	0.51	2.15
NH_3_	0.87	2.70	1.13	7.04
Asp	0.12	0.06	0.16	0.16
Thr	5.40	2.10	7.03	5.48
Ser	1.86	0.96	2.42	2.50
Glu	2.40	1.71	3.13	4.46
sarcosine	-	0.98	-	2.57
Gly	14.37	3.30	18.72	8.63
Ala	4.54	0.74	5.91	1.93
Val	4.40	2.09	5.73	5.45
Cys	-	0.36	-	0.94
Ile	3.40	1.26	4.43	3.28
Leu	4.62	1.44	6.02	3.75
Tyr	4.48	1.06	5.84	2.76
Phe	2.89	2.04	3.76	5.34
β-Ala	0.15	0.04	0.20	0.10
β-amino isobutyric acid	-	0.20	-	0.52
GABA	0.26	0.07	0.34	0.17
EtAm	0.82	0.64	1.07	1.67
Orn	0.82	0.57	1.07	1.50
Lys	6.48	3.78	8.44	9.88
His	1.94	1.64	2.53	4.28
3-MeHis	0.36	0.12	0.47	0.31
Arg	2.64	0.74	3.44	1.92
hydroxy proline	-	0.16	-	0.41
Pro	13.55	8.65	17.65	22.58
Total	76.76	38.29	100.00	100.00

^a^ Amino acid contents in larval saliva of *V. mandarinia* is quoted from the previous research by Takashi et al. [27].

**Table 3:** Hydrolyzed amino acid compositions of larval saliva of *V. v. nigrithorax* compared to that of *V. mandarinia*.

Amino Acids	*V. mandarinia*	*V. v. nigrithorax*	*V. mandarinia*	*V. v. nigrithorax*
	μmol/mL ^a^	μmol/mL	mol% ^a^	mol%
Asp	12.29	3.21	5.62	4.77
Thr	6.71	3.18	3.07	4.74
Ser	8.47	1.59	3.87	2.36
Glu	32.17	10.71	14.71	15.93
Gly	29.85	8.24	13.65	12.25
Ala	8.90	2.14	4.07	3.18
Cys	0.67	0.57	0.31	0.85
Val	11.96	4.26	5.47	6.34
Met	3.15	0.33	1.44	0.49
Ile	15.27	3.29	6.98	4.90
Leu	15.98	3.44	7.31	5.11
Tyr	7.74	0.76	3.54	1.13
Phe	6.12	3.45	2.80	5.13
Lys	22.81	7.29	10.43	10.85
His	14.13	4.00	6.46	5.96
Arg	12.75	1.60	5.83	2.38
Pro	9.72	9.17	4.44	13.64
Total	218.69	67.24	100.00	100.00

^a^ Amino acid contents in larval saliva of *V. mandarinia* is quoted from the previous research by Takashi et al. [27].

**Table 4:** Nutritional composition of *V. v. nigrithorax* larvae, compared to those of *V. mandarinia, P. b. seulensis* and *T. molitor* larvae (g/100 g).

Nutrients	*Vespa velutina nigrithorax*	*Vespa mandarinia* ^a^	*Protaetia brevitarsis seulensis* ^a^	*Tenebrio molitor* ^a^
Moisture	2.78	-	6.66	5.33
Crude protein	48.64	59.7	57.86	46.44
Crude fat	13.23	20.6	16.57	32.70
Crude ash	3.04	4.1	8.36	2.86
Carbohydrate	32.31	15.6	10.56	12.67

^a^ Nutritional composition of *V. mandarinia* [13], *P. b. seulensis* [40] and *T. molitor* [41] larvae are quoted from previous research.

**Table 5:** Amino acids composition in the larvae.

Amino Acids	Free Amino Acids in The Larvae (mg/g)	Free Amino Acids in the Larvae (mol%)	Hydrolyzed Amino Acids in the Larvae (mg/g)	Hydrolyzed Amino Acids in the Larvae (mol%)
phosphoserine	0.63	0.53		
Tau	8.14	10.17		
NH_3_	0.30	2.76		
Asp	0.45	0.53	24.86	7.38
Thr	3.17	4.16	13.35	4.43
Ser	1.59	2.36	11.37	4.28
Glu	2.87	3.05	44.71	12.02
Gly	10.26	21.37	17.51	9.22
Ala	2.85	5.00	12.12	5.38
Val	2.79	3.72	18.08	6.10
Cys	-	-	3.31	1.08
Ile	2.68	3.19	13.61	4.10
Leu	3.31	3.95	21.81	6.57
Tyr	3.62	3.12	12.07	2.63
Phe	2.68	2.54	12.90	3.09
β-Ala	0.56	0.99		
β-amino isobutyric acid	0.30	0.46		
EtAm	0.15	0.51		
Orn	0.62	0.73		
Lys	5.17	5.53	21.79	5.89
His	1.29	1.30	7.74	3.23
3-MeHis	0.10	0.10		
Arg	6.43	5.77	14.21	1.97
hydroxy proline	0.37	0.44		
Pro	12.24	16.62	24.58	13.21
Total	74.41	100	277.68	100

**Table 6:** Comparison of the essential amino acids amounts required for an adult per day and amino acids contained in 100 g of *V. v. nigrithorax* larvae.

Amino Acids	Daily Adult Amino Acid Requirements (mg/kg) ^a^	Daily Amino Acid Requirements for a 70-kg Adult (mg)	Amino Acid Provided by 100 g of *V. v. nigrithorax* Larvae (mg) ^b^	Ratio of Amino Acid Provided to Requirements (%)
Thr	15.0	1050	1335	127
Val	39.0	1820	1808	99
Cys	4.1	287	331	115
Ile	20.0	1400	1361	97
Leu	39.0	2730	2181	80
Phe + Tyr	25.0	1750	2497	143
Lys	30.0	2100	2179	104
His	10.0	700	774	111
Trp	4.0	280	-	
Met	10.4	728	-	

^a^ Based on the daily amino acids requirements for an adult suggested by the WHO (World Health Organization) [47]; ^b^ deduced from the amino acid contents in the hydrolyzed product of the larvae in Table 5.
